# (*E*)-*N*′-(4-Meth­oxy­benzyl­idene)-2-*m*-tolyl­acetohydrazide

**DOI:** 10.1107/S1600536812047113

**Published:** 2012-11-24

**Authors:** A. S. Praveen, Jerry P. Jasinski, Amanda C. Keeley, H. S. Yathirajan, B. Narayana

**Affiliations:** a Department of Studies in Chemistry, University of Mysore, Manasagangotri, Mysore 570 006, India; b Department of Chemistry, Keene State College, 229 Main Street, Keene, NH 03435-2001, USA; c Department of Studies in Chemistry, Mangalore University, Mangalagangotri 574 199, India

## Abstract

In the title mol­ecule, C_17_H_18_N_2_O_2_, the benzene rings form a dihedral angle of 83.0 (7)°. In the crystal, N—H⋯O hydrogen bonds, in an *R* ^2^_2_(8) graph-set motif, link mol­ecules into centrocymmetric dimers, and weak C—H⋯π inter­actions further link these dimers into columns in [100].

## Related literature

For the biological activity of Schiff bases, see: Desai *et al.* (2001 ▶); El-Masry *et al.* (2000 ▶); Hodnett & Dunn (1970 ▶); Pandey *et al.* (1999 ▶); Singh & Dash (1988 ▶). For Schiff bases employed as ligands for complexation of metal ions, see: Aydogan *et al.* (2001 ▶). For Schiff bases with applications in dyes and pigments, see: Taggi *et al.* (2002 ▶). For related structures, see: Akkurt *et al.* (2011 ▶); Lv *et al.* (2009*a* ▶,*b* ▶); Yu & Lv (2010 ▶). For standard bond lengths, see: Allen *et al.* (1987 ▶).
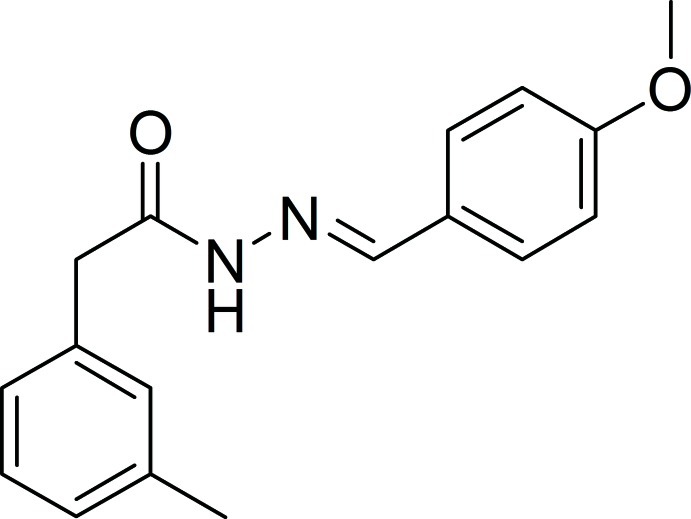


## Experimental

### 

#### Crystal data

C_17_H_18_N_2_O_2_
*M* *_r_* = 282.33
Triclinic, 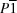

*a* = 6.4961 (8) Å
*b* = 9.8047 (10) Å
*c* = 12.7464 (13) Å
α = 112.130 (9)°
β = 95.507 (10)°
γ = 96.601 (9)°
*V* = 738.45 (14) Å^3^
*Z* = 2
Cu *K*α radiation
μ = 0.68 mm^−1^
*T* = 173 K
0.34 × 0.14 × 0.06 mm

#### Data collection

Oxford Diffraction Xcalibur Eos Gemini diffractometer
Absorption correction: multi-scan (*CrysAlis PRO*; Oxford Diffraction, 2010 ▶) *T*_min_ = 0.735, *T*_max_ = 1.000
4418 measured reflections
2840 independent reflections
2032 reflections with *I* > 2σ(*I*)
*R*_int_ = 0.034

#### Refinement

*R*[*F* ^2^ > 2σ(*F* ^2^)] = 0.052
*wR*(*F* ^2^) = 0.158
*S* = 1.04
2840 reflections
193 parameters
H-atom parameters constrained
Δρ_max_ = 0.21 e Å^−3^
Δρ_min_ = −0.20 e Å^−3^

### 

Data collection: *CrysAlis PRO* (Oxford Diffraction, 2010 ▶); cell refinement: *CrysAlis PRO*; data reduction: *CrysAlis RED* (Oxford Diffraction, 2010 ▶); program(s) used to solve structure: *SHELXS97* (Sheldrick, 2008 ▶); program(s) used to refine structure: *SHELXL97* (Sheldrick, 2008 ▶); molecular graphics: *SHELXTL* (Sheldrick, 2008 ▶); software used to prepare material for publication: *SHELXTL*.

## Supplementary Material

Crystal structure: contains datablock(s) I. DOI: 10.1107/S1600536812047113/cv5364sup1.cif

Structure factors: contains datablock(s) I. DOI: 10.1107/S1600536812047113/cv5364Isup2.hkl

Supplementary material file. DOI: 10.1107/S1600536812047113/cv5364Isup3.cml

Additional supplementary materials:  crystallographic information; 3D view; checkCIF report

## Figures and Tables

**Table 1:** Hydrogen-bond geometry (Å, °) *Cg* is the centroid of the C3–C8 ring.

*D*—H⋯*A*	*D*—H	H⋯*A*	*D*⋯*A*	*D*—H⋯*A*
N1—H1⋯O1^i^	0.86	2.04	2.902 (2)	178
C15—H15⋯*Cg* ^ii^	0.93	2.63	3.557 (2)	173

Symmetry codes: (i) 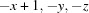; (ii) 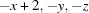.

**Fig. 1.**
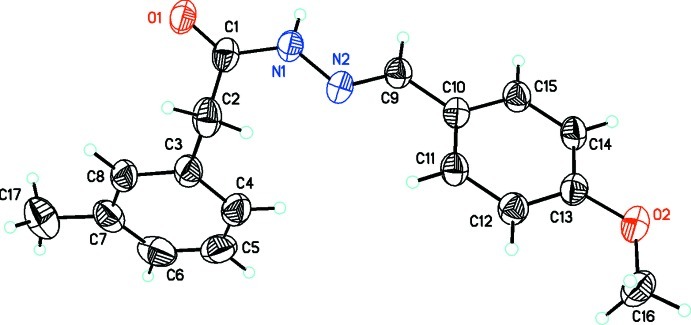
Molecular structure of the title compound showing the atom labeling scheme and 50% probability displacement ellipsoids.

**Fig. 2.**
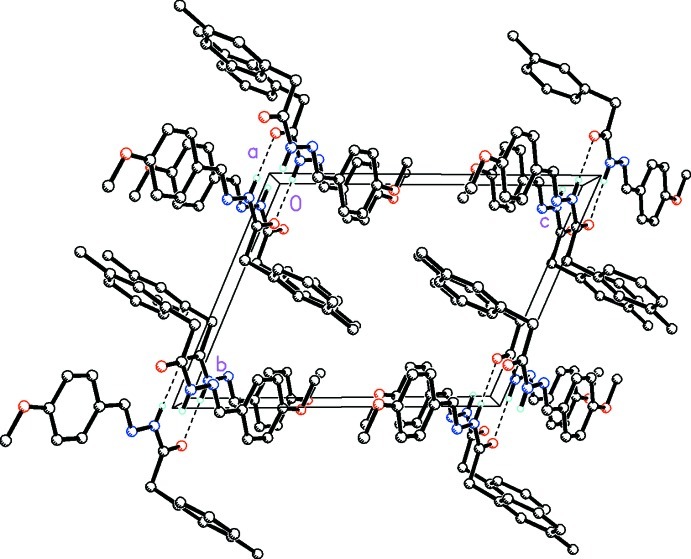
Packing diagram of the title compound viewed along the a axis. Dashed lines indicate N—H···O hydrogen bonds. The remaining H atoms have been removed for clarity.

**Fig. 3.**
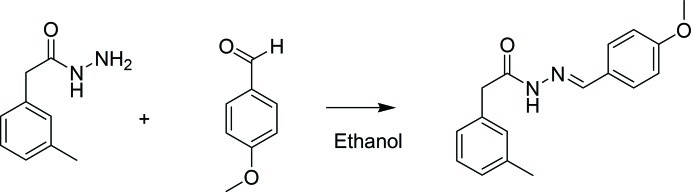
Synthesis of the title compound.

## supplementary crystallographic information

### Comment

Schiff bases are known to have biological activities such as antimicrobial (El-Masry *et al.*, 2000; Pandey *et al.*, 1999), antifungal (Singh *et al.*, 1988), antitumor (Hodnett *et al.*, 1970; Desai *et al.*, 2001), and as herbicides. Schiff bases have also been employed as ligands for complexation of metal ions (Aydogan *et al.*, 2001). On the industrial scale, they have wide range of applications such as dyes and pigments (Taggi *et al.*, 2002). The crystal structures of some Schiff base hydrazines, viz., N'-(2-methoxybenzylidene) acetohydrazide (Yu & Lv, 2010), 2-[6-(4-chlorophenyl)imidazo[2,1-b][1,3] thiazol-2-yl]-N'-[(E)-4-methoxybenzylidene]acetohydrazide (Akkurt *et al.*, 2011), N'-(3-methoxybenzylidene)acetohydrazide and N'-(3,4-dimethoxybenzylidene)acetohydrazide (Lv *et al.*, 2009*a*,*b*). In view of the importance of hydrazides, the crystal structure of title compound (I) is reported.

In the title molecule, C_17_H_18_N_2_O_2_, two benzene rings form a dihedral angle of 83.0 (7)° (Fig. 1). Bond lengths are in normal ranges (Allen, 1987). In the crystal, N—H···O hydrogen bonds (Table 1), in an *R*^2^_2_(8) graph set motif, link molecules into centrocymmetric dimers, and weak C–H···π interactions (Table 1) link further these dimers into columns in [100] (Fig. 2).

### Experimental

To a stirred solution of 2-m-tolylacetohydrazide (1 g, 6.09 mmol) in ethanol (10 mL), 4-methoxybenzaldehyde (0.79 g, 6.09 mmol) was added (Fig. 3) and strirred at room temperature for 30 minutes. Precipitated solid was filtered and dried. The single crystal was grown from ethyl acetate by slow evaporation method and yield of the compound was 94% (m.p.: 403-405 K).

### Refinement

All H atoms were placed in their calculated positions and then refined using the riding model with Atom—H lengths of 0.93Å (CH), 0.97Å (CH_2_), 0.96Å (CH_3_) or 0.86Å (NH). Isotropic displacement parameters for these atoms were set to 1.19-1.21 (CH, CH_2_), 1.49 (CH_3_) or 1.21 (NH) times *U*_eq_ of the parent atom.

### Crystal data

**Table:**

C_17_H_18_N_2_O_2_	*Z* = 2
*M**_r_* = 282.33	*F*(000) = 300
Triclinic, *P*1	*D*_x_ = 1.270 Mg m^−^^3^
Hall symbol: -P 1	Cu *K*α radiation, λ = 1.54184 Å
*a* = 6.4961 (8) Å	Cell parameters from 1237 reflections
*b* = 9.8047 (10) Å	θ = 3.8–72.4°
*c* = 12.7464 (13) Å	µ = 0.68 mm^−^^1^
α = 112.130 (9)°	*T* = 173 K
β = 95.507 (10)°	Chunk, colorless
γ = 96.601 (9)°	0.34 × 0.14 × 0.06 mm
*V* = 738.45 (14) Å^3^	

### Data collection

**Table:**

Oxford Diffraction Xcalibur Eos Gemini diffractometer	2840 independent reflections
Radiation source: Enhance (Cu) X-ray Source	2032 reflections with *I* > 2σ(*I*)
Graphite monochromator	*R*_int_ = 0.034
Detector resolution: 16.0416 pixels mm^-1^	θ_max_ = 72.6°, θ_min_ = 3.8°
ω scans	*h* = −8→7
Absorption correction: multi-scan (*CrysAlis PRO*; Oxford Diffraction, 2010)	*k* = −12→8
*T*_min_ = 0.735, *T*_max_ = 1.000	*l* = −11→15
4418 measured reflections	

### Refinement

**Table:**

Refinement on *F*^2^	Secondary atom site location: difference Fourier map
Least-squares matrix: full	Hydrogen site location: inferred from neighbouring sites
*R*[*F*^2^ > 2σ(*F*^2^)] = 0.052	H-atom parameters constrained
*wR*(*F*^2^) = 0.158	*w* = 1/[σ^2^(*F*_o_^2^) + (0.064*P*)^2^ + 0.071*P*] where *P* = (*F*_o_^2^ + 2*F*_c_^2^)/3
*S* = 1.04	(Δ/σ)_max_ < 0.001
2840 reflections	Δρ_max_ = 0.21 e Å^−^^3^
193 parameters	Δρ_min_ = −0.20 e Å^−^^3^
0 restraints	Extinction correction: *SHELXL*, Fc^*^=kFc[1+0.001xFc^2^λ^3^/sin(2θ)]^-1/4^
Primary atom site location: structure-invariant direct methods	Extinction coefficient: 0.0097 (12)

### Special details

**Table:**

Geometry. All esds (except the esd in the dihedral angle between two l.s. planes) are estimated using the full covariance matrix. The cell esds are taken into account individually in the estimation of esds in distances, angles and torsion angles; correlations between esds in cell parameters are only used when they are defined by crystal symmetry. An approximate (isotropic) treatment of cell esds is used for estimating esds involving l.s. planes.
Refinement. Refinement of *F*^2^ against ALL reflections. The weighted *R*-factor *wR* and goodness of fit *S* are based on *F*^2^, conventional *R*-factors *R* are based on *F*, with *F* set to zero for negative *F*^2^. The threshold expression of *F*^2^ > σ(*F*^2^) is used only for calculating *R*-factors(gt) *etc*. and is not relevant to the choice of reflections for refinement. *R*-factors based on *F*^2^ are statistically about twice as large as those based on *F*, and *R*- factors based on ALL data will be even larger.

### Fractional atomic coordinates and isotropic or equivalent isotropic displacement parameters (Å^2^)

**Table:**

	*x*	*y*	*z*	*U*_iso_*/*U*_eq_	
O1	0.4694 (2)	0.19694 (16)	0.05426 (13)	0.0429 (4)	
O2	1.6491 (2)	−0.04156 (17)	−0.38486 (13)	0.0482 (4)	
N1	0.7161 (2)	0.09164 (19)	−0.04553 (14)	0.0363 (4)	
H1	0.6645	0.0052	−0.0486	0.044*	
N2	0.8879 (2)	0.10557 (19)	−0.09800 (14)	0.0365 (4)	
C1	0.6266 (3)	0.2111 (2)	0.01087 (17)	0.0376 (5)	
C2	0.7332 (4)	0.3634 (2)	0.02237 (19)	0.0447 (5)	
H2A	0.8138	0.3528	−0.0397	0.054*	
H2B	0.6288	0.4255	0.0188	0.054*	
C3	0.8763 (3)	0.4349 (2)	0.13628 (19)	0.0397 (5)	
C4	1.0802 (4)	0.4059 (2)	0.1471 (2)	0.0488 (6)	
H4	1.1354	0.3504	0.0826	0.059*	
C5	1.2004 (4)	0.4600 (3)	0.2543 (3)	0.0584 (7)	
H5	1.3365	0.4402	0.2617	0.070*	
C6	1.1199 (4)	0.5432 (3)	0.3507 (3)	0.0596 (7)	
H6	1.2023	0.5784	0.4222	0.071*	
C7	0.9182 (4)	0.5748 (2)	0.3419 (2)	0.0487 (6)	
C8	0.7994 (3)	0.5198 (2)	0.2336 (2)	0.0419 (5)	
H8	0.6640	0.5408	0.2262	0.050*	
C9	0.9579 (3)	−0.0161 (2)	−0.14981 (17)	0.0353 (5)	
H9	0.8927	−0.1052	−0.1490	0.042*	
C10	1.1381 (3)	−0.0163 (2)	−0.20977 (16)	0.0336 (4)	
C11	1.2404 (3)	0.1120 (2)	−0.21651 (17)	0.0365 (5)	
H11	1.1929	0.2020	−0.1818	0.044*	
C12	1.4114 (3)	0.1087 (2)	−0.27377 (18)	0.0376 (5)	
H12	1.4781	0.1958	−0.2771	0.045*	
C13	1.4832 (3)	−0.0259 (2)	−0.32644 (17)	0.0362 (5)	
C14	1.3827 (3)	−0.1543 (2)	−0.31983 (18)	0.0403 (5)	
H14	1.4310	−0.2441	−0.3541	0.048*	
C15	1.2124 (3)	−0.1503 (2)	−0.26320 (17)	0.0379 (5)	
H15	1.1458	−0.2377	−0.2603	0.045*	
C16	1.7630 (3)	0.0894 (3)	−0.3878 (2)	0.0523 (6)	
H16A	1.6733	0.1318	−0.4272	0.078*	
H16B	1.8807	0.0643	−0.4272	0.078*	
H16C	1.8118	0.1605	−0.3109	0.078*	
C17	0.8251 (5)	0.6648 (3)	0.4450 (2)	0.0709 (8)	
H17A	0.7722	0.7457	0.4325	0.106*	
H17B	0.9312	0.7039	0.5114	0.106*	
H17C	0.7128	0.6020	0.4568	0.106*	

### Atomic displacement parameters (Å^2^)

**Table:**

	*U*^11^	*U*^22^	*U*^33^	*U*^12^	*U*^13^	*U*^23^
O1	0.0382 (8)	0.0476 (9)	0.0442 (8)	0.0160 (7)	0.0149 (7)	0.0148 (7)
O2	0.0452 (9)	0.0510 (10)	0.0483 (9)	0.0128 (7)	0.0216 (7)	0.0144 (7)
N1	0.0370 (9)	0.0360 (9)	0.0391 (9)	0.0108 (7)	0.0137 (7)	0.0150 (7)
N2	0.0349 (9)	0.0411 (10)	0.0373 (9)	0.0126 (7)	0.0123 (7)	0.0163 (8)
C1	0.0375 (11)	0.0448 (12)	0.0347 (10)	0.0177 (9)	0.0081 (9)	0.0164 (9)
C2	0.0544 (13)	0.0416 (12)	0.0501 (13)	0.0228 (10)	0.0188 (10)	0.0245 (10)
C3	0.0438 (12)	0.0301 (10)	0.0523 (13)	0.0117 (9)	0.0164 (10)	0.0204 (9)
C4	0.0453 (13)	0.0380 (12)	0.0692 (16)	0.0117 (10)	0.0231 (12)	0.0232 (11)
C5	0.0348 (12)	0.0457 (14)	0.096 (2)	0.0049 (10)	0.0052 (13)	0.0307 (14)
C6	0.0587 (16)	0.0412 (13)	0.0705 (18)	−0.0021 (11)	−0.0082 (13)	0.0199 (12)
C7	0.0599 (14)	0.0317 (11)	0.0513 (14)	0.0062 (10)	0.0098 (11)	0.0127 (10)
C8	0.0418 (12)	0.0312 (10)	0.0584 (14)	0.0134 (9)	0.0173 (10)	0.0194 (10)
C9	0.0378 (11)	0.0337 (10)	0.0366 (10)	0.0095 (8)	0.0089 (8)	0.0144 (8)
C10	0.0347 (10)	0.0361 (10)	0.0310 (10)	0.0106 (8)	0.0071 (8)	0.0126 (8)
C11	0.0397 (11)	0.0330 (10)	0.0391 (11)	0.0134 (8)	0.0097 (9)	0.0135 (9)
C12	0.0389 (11)	0.0360 (11)	0.0403 (11)	0.0089 (8)	0.0086 (9)	0.0160 (9)
C13	0.0348 (10)	0.0428 (11)	0.0310 (10)	0.0098 (8)	0.0075 (8)	0.0130 (9)
C14	0.0429 (12)	0.0357 (11)	0.0405 (11)	0.0149 (9)	0.0126 (9)	0.0091 (9)
C15	0.0426 (11)	0.0320 (10)	0.0400 (11)	0.0095 (8)	0.0103 (9)	0.0132 (9)
C16	0.0429 (13)	0.0643 (16)	0.0530 (14)	0.0051 (11)	0.0186 (11)	0.0249 (12)
C17	0.096 (2)	0.0541 (16)	0.0542 (16)	0.0169 (15)	0.0162 (15)	0.0096 (13)

### Geometric parameters (Å, º)

**Table:**

O1—C1	1.225 (2)	C7—C17	1.508 (3)
O2—C13	1.358 (2)	C8—H8	0.9300
O2—C16	1.422 (3)	C9—C10	1.457 (3)
N1—C1	1.352 (2)	C9—H9	0.9300
N1—N2	1.374 (2)	C10—C11	1.390 (3)
N1—H1	0.8600	C10—C15	1.399 (3)
N2—C9	1.286 (2)	C11—C12	1.383 (3)
C1—C2	1.520 (3)	C11—H11	0.9300
C2—C3	1.513 (3)	C12—C13	1.394 (3)
C2—H2A	0.9700	C12—H12	0.9300
C2—H2B	0.9700	C13—C14	1.386 (3)
C3—C8	1.385 (3)	C14—C15	1.374 (3)
C3—C4	1.391 (3)	C14—H14	0.9300
C4—C5	1.383 (4)	C15—H15	0.9300
C4—H4	0.9300	C16—H16A	0.9600
C5—C6	1.383 (4)	C16—H16B	0.9600
C5—H5	0.9300	C16—H16C	0.9600
C6—C7	1.384 (3)	C17—H17A	0.9600
C6—H6	0.9300	C17—H17B	0.9600
C7—C8	1.389 (3)	C17—H17C	0.9600
			
C13—O2—C16	117.66 (17)	N2—C9—H9	119.5
C1—N1—N2	121.37 (17)	C10—C9—H9	119.5
C1—N1—H1	119.3	C11—C10—C15	118.04 (18)
N2—N1—H1	119.3	C11—C10—C9	122.64 (18)
C9—N2—N1	115.66 (17)	C15—C10—C9	119.32 (18)
O1—C1—N1	121.0 (2)	C12—C11—C10	121.44 (18)
O1—C1—C2	121.47 (18)	C12—C11—H11	119.3
N1—C1—C2	117.52 (18)	C10—C11—H11	119.3
C3—C2—C1	108.30 (17)	C11—C12—C13	119.67 (19)
C3—C2—H2A	110.0	C11—C12—H12	120.2
C1—C2—H2A	110.0	C13—C12—H12	120.2
C3—C2—H2B	110.0	O2—C13—C14	116.24 (18)
C1—C2—H2B	110.0	O2—C13—C12	124.44 (19)
H2A—C2—H2B	108.4	C14—C13—C12	119.32 (19)
C8—C3—C4	118.9 (2)	C15—C14—C13	120.69 (19)
C8—C3—C2	119.98 (19)	C15—C14—H14	119.7
C4—C3—C2	120.8 (2)	C13—C14—H14	119.7
C5—C4—C3	119.6 (2)	C14—C15—C10	120.84 (19)
C5—C4—H4	120.2	C14—C15—H15	119.6
C3—C4—H4	120.2	C10—C15—H15	119.6
C4—C5—C6	120.6 (2)	O2—C16—H16A	109.5
C4—C5—H5	119.7	O2—C16—H16B	109.5
C6—C5—H5	119.7	H16A—C16—H16B	109.5
C5—C6—C7	120.8 (3)	O2—C16—H16C	109.5
C5—C6—H6	119.6	H16A—C16—H16C	109.5
C7—C6—H6	119.6	H16B—C16—H16C	109.5
C6—C7—C8	117.9 (2)	C7—C17—H17A	109.5
C6—C7—C17	122.3 (3)	C7—C17—H17B	109.5
C8—C7—C17	119.8 (2)	H17A—C17—H17B	109.5
C3—C8—C7	122.1 (2)	C7—C17—H17C	109.5
C3—C8—H8	119.0	H17A—C17—H17C	109.5
C7—C8—H8	119.0	H17B—C17—H17C	109.5
N2—C9—C10	120.92 (18)		
			
C1—N1—N2—C9	−179.39 (18)	C17—C7—C8—C3	−179.1 (2)
N2—N1—C1—O1	177.62 (17)	N1—N2—C9—C10	179.35 (16)
N2—N1—C1—C2	−4.8 (3)	N2—C9—C10—C11	−1.4 (3)
O1—C1—C2—C3	83.1 (2)	N2—C9—C10—C15	178.72 (18)
N1—C1—C2—C3	−94.5 (2)	C15—C10—C11—C12	−0.2 (3)
C1—C2—C3—C8	−88.4 (2)	C9—C10—C11—C12	179.85 (18)
C1—C2—C3—C4	85.9 (2)	C10—C11—C12—C13	0.2 (3)
C8—C3—C4—C5	1.0 (3)	C16—O2—C13—C14	−176.56 (19)
C2—C3—C4—C5	−173.3 (2)	C16—O2—C13—C12	3.1 (3)
C3—C4—C5—C6	−0.3 (3)	C11—C12—C13—O2	179.91 (18)
C4—C5—C6—C7	−0.3 (4)	C11—C12—C13—C14	−0.5 (3)
C5—C6—C7—C8	0.3 (4)	O2—C13—C14—C15	−179.62 (18)
C5—C6—C7—C17	179.8 (2)	C12—C13—C14—C15	0.7 (3)
C4—C3—C8—C7	−1.2 (3)	C13—C14—C15—C10	−0.7 (3)
C2—C3—C8—C7	173.23 (19)	C11—C10—C15—C14	0.5 (3)
C6—C7—C8—C3	0.5 (3)	C9—C10—C15—C14	−179.58 (18)

### Hydrogen-bond geometry (Å, º)

Cg is the centroid of the C3–C8 ring.

**Table:**

*D*—H···*A*	*D*—H	H···*A*	*D*···*A*	*D*—H···*A*
N1—H1···O1^i^	0.86	2.04	2.902 (2)	178
C15—H15···*Cg*^ii^	0.93	2.63	3.557 (2)	173

Symmetry codes: (i) −*x*+1, −*y*, −*z*; (ii) −*x*+2, −*y*, −*z*.

## References

[bb1] Akkurt, M., Güzeldemirci, N. U., Karaman, B. & Büyükgüngör, O. (2011). *Acta Cryst.* E**67**, o184–o185. 10.1107/S1600536810052359 PMC3050215 21522689

[bb2] Allen, F. H., Kennard, O., Watson, D. G., Brammer, L., Orpen, A. G. & Taylor, R. (1987). *J. Chem. Soc. Perkin Trans. 2*, pp. S1–19.

[bb3] Aydogan, F., Ocal, N., Turgut, Z. & Yolacan, C. (2001). *Bull. Korean Chem. Soc.* **22**, 476–480.

[bb4] Desai, S. B., Desai, P. B. & Desai, K. R. (2001). *Heterocycl. Commun.* **7**, 83–90.

[bb5] El-Masry, A. H., Fahmy, H. H. & Abdelwahed, S. H. A. (2000). *Molecules*, **5**, 1429—1438.

[bb6] Hodnett, E. M. & Dunn, W. J. (1970). *J. Med. Chem.* **13**, 768–770. 10.1021/jm00298a054 5452451

[bb7] Lv, L.-P., Yu, T.-M., Yu, W.-B., Li, W.-W. & Hu, X.-C. (2009*a*). *Acta Cryst.* E**65**, o1990. 10.1107/S1600536809028864 PMC2977277 21583664

[bb8] Lv, L.-P., Yu, T.-M., Yu, W.-B., Li, W.-W. & Hu, X.-C. (2009*b*). *Acta Cryst.* E**65**, o1989. 10.1107/S1600536809027366 PMC2977411 21583663

[bb9] Oxford Diffraction (2010). *CrysAlis PRO* and *CrysAlis RED* Oxford Diffraction Ltd, Abingdon, England.

[bb10] Pandey, S. N., Sriram, D., Nath, G. & De Clercq, E. (1999). *Il Farmaco*, **54**, 624–628. 10.1016/s0014-827x(99)00075-0 10555264

[bb11] Sheldrick, G. M. (2008). *Acta Cryst.* A**64**, 112–122. 10.1107/S0108767307043930 18156677

[bb12] Singh, W. M. & Dash, B. C. (1988). *Pesticides*, **22**, 33–37.

[bb13] Taggi, A. E., Hafez, A. M., Wack, H., Young, B., Ferraris, D. & Lectka, T. (2002). *J. Am. Chem. Soc.* **124**, 6626–6635. 10.1021/ja0258226 12047183

[bb14] Yu, T.-M. & Lv, L.-P. (2010). *Acta Cryst.* E**66**, o2666. 10.1107/S1600536810038092 PMC2983360 21587637

